# Outdoor play among children in relation to neighborhood characteristics: a cross-sectional neighborhood observation study

**DOI:** 10.1186/1479-5868-9-98

**Published:** 2012-08-17

**Authors:** Marie-Jeanne Aarts, Sanne I de Vries, Hans AM van Oers, Albertine J Schuit

**Affiliations:** 1 Tilburg School of Social and Behavioral Sciences, Department Tranzo, Scientific Center for Care and Welfare, Tilburg University, PO Box 90153, 5000, LE, Tilburg, The Netherlands; 2 National Institute for Public Health and the Environment, Public Health and Health Services Division, PO Box 1, 3720, BA, Bilthoven, The Netherlands; 3 CAPHRI School for Public Health and Primary Care, Department of Health Services Research, Maastricht University, PO Box 616, 6200, MD, Maastricht, The Netherlands; 4 TNO, Department of Life Style, PO Box 2215, 2301, CE, Leiden, The Netherlands; 5 Department of Health Sciences and EMGO Institute for Health and Care Research, VU University Amsterdam, De Boelelaan 1105, 1081, HV, Amsterdam, The Netherlands

**Keywords:** Physical activity, Children, Outdoor play, Environment, Neighborhood observations

## Abstract

**Background:**

Although environmental characteristics as perceived by parents are known to be related to children’s outdoor play behavior, less is known about the relation between independently measured neighborhood characteristics and outdoor play among children. The purpose of this study was to identify quantitative as well as qualitative neighborhood characteristics related to outdoor play by means of neighborhood observations.

**Methods:**

Questionnaires including questions on outdoor play behavior of the child were distributed among 3,651 parents of primary school children (aged 4–12 years). Furthermore, neighborhood observations were conducted in 33 Dutch neighborhoods to map neighborhood characteristics such as buildings, formal outdoor play facilities, public space, street pattern, traffic safety, social neighborhood characteristics, and general impression. Data of the questionnaires and the neighborhood observations were coupled via postal code of the respondents. Multilevel GEE analyses were performed to quantify the correlation between outdoor play and independently measured neighborhood characteristics.

**Results:**

Parental education was negatively associated with outdoor play among children. Neither the presence nor the overall quality of formal outdoor play facilities were (positively) related to outdoor play among children in this study. Rather, informal play areas such as the presence of sidewalks were related to children’s outdoor play. Also, traffic safety was an important characteristic associated with outdoor play.

**Conclusions:**

This study showed that, apart from individual factors such as parental education level, certain modifiable characteristics in the neighborhood environment (as measured by neighborhood observations) were associated with outdoor play among boys and girls of different age groups in The Netherlands. Local policy makers from different sectors can use these research findings in creating more activity-friendly neighborhoods for children.

## Background

As in many other Western countries, the majority of primary school children in The Netherlands does not meet the recommended guidelines for health enhancing physical activity [1]. It is therefore important to find appropriate ways to stimulate physical activity among children, for example by stimulating outdoor play [2]. Time spent outdoors is consistently related to children’s physical activity level [3-7] and hence stimulating outdoor play among children may contribute to their physical and mental health. A recent study by Kimbro et al. has shown for example that time spent on outdoor play was associated with lower BMI values among children [8].

Research has shown that environmental characteristics can play a role in children’s physical activity [5,9]. More specifically, in a previous study we have shown that both the perceived physical environment and the perceived social environment were related to children’s outdoor play [10]. This study showed that children of parents that perceive a high social cohesion in their neighborhood spend more time on outdoor play. Regarding the built environment, the study showed that different environmental characteristics are related to children of different age groups.

Research among adults [11-13] and adolescents [14,15] has shown that differences exist in correlates of physical activity when measured subjectively (i.e. perceived environmental characteristics as measured with questionnaires among parents), independently (i.e. by neighborhood observations by independent observers), or objectively (i.e. by means of geographical information systems).

A previous Dutch study has investigated the role of independently measured physical environmental characteristics in disadvantaged neighborhoods on physical activity among children aged 6 to 11 years by means of neighborhood observations [16]. The authors conclude that children’s physical activity is indeed associated with certain modifiable factors of the built environment, such as parking spaces in the neighborhood. The authors further stress the specificity of environmental characteristics in relation to different behavioral components (e.g. moderate-to-vigorous physical activity, walking, bicycling etc.) [17], a point also mentioned by others [18].

While the abovementioned Dutch study mainly described the quantitative aspects of the built environment (i.e. the absence, presence or amount of neighborhood facilities), it is assumed that the quality of neighborhood facilities (e.g. the accessibility and state of maintenance) may be important in relation to children’s physical activity level as well. Therefore, the aim of the present study was to identify quantitative as well as qualitative neighborhood characteristics related to outdoor play among primary school children (aged 4 to 12 years) by means of neighborhood observations.

## Methods

### Study design and setting

This cross-sectional study was situated in four medium-sized cities in the Southern part of The Netherlands. The number of inhabitants ranged from 77,450 to 201,259 and the population density ranged from 727 to 1,716 citizens per km^2^. Although one city was somewhat smaller and less urbanized compared to the other cities, they were comparable regarding the demography of their population such as the percentage of non-Western immigrants (range: 9.9 - 13.4%) and the percentage of inhabitants aged 0–14 years (range: 16.7-17.6%). The selection procedures and characteristics of the participating cities are described in more detail elsewhere [19].

Data on physical activity behavior of the children were obtained by means of a cross-sectional survey consisting of a written questionnaire for parents between September 2007 and January 2008. The data on neighborhood characteristics were collected approximately one year later (between October and December 2008) by means of standardized neighborhood observations (audits) by trained observers. Based on postal code (six positions: four numbers, two letters available from both the questionnaire among parents and from municipal data describing which postal codes fall within one neighborhood), the data from these two study parts were combined for the analyses of this paper. Both study parts will be described in more detail below.

### Survey among parents

The study was targeted at primary school children aged 4–12 years. In the Netherlands, children in this age group attend primary school, which, in most cases, is close to or within the area of residence. Initially, all regular primary schools in the four cities (n = 149), except those already participating in other (research) projects aimed at physical activity among children (n = 34) were invited by letter, followed up by a telephone call to participate in the survey. Of the invited schools (n = 115), approximately one third agreed to participate (n = 42). As outlined elsewhere [19], the schools in our study were representative for the total population of schools in the participating cities in terms of school size, socioeconomic status and type of neighborhood.

At each school enrolled in the study, all grades and classes were included in the survey. Because no medical or physical measurements were conducted and considering the negligible (psychological) burden to fill in the questionnaire, no ethics approval was required according to the Dutch Central Committee on Research Investigating Human Subjects. Parents were given written information about the study and by returning the questionnaire they gave consent for the inclusion of their data in the study. In total parents of 11,094 children were provided with a questionnaire. Parents that had more than one child attending the same school, were provided with a questionnaire for each individual child. Response rate was 60%, resulting in 6,624 returned questionnaires. During data entry, 12 questionnaires could not be read and 11 questionnaires were removed because they were completely empty, leaving 6,601 completed and returned questionnaires.

Parents were asked to report the frequency (number of school days and number of days per weekend) their child was involved in outdoor play, considering a typical week in the past month. Parents were also asked to report the duration of outdoor play during week and weekend days (less than 30 minutes per day, 30 minutes to one hour per day, one to two hours per day, more than two hours per day). Furthermore, the questionnaire included items on age and gender of the child and parental education level and net household income per month. Based on parental report of weight and height of their child, BMI was calculated and percentage overweight and obesity (as determined by age and gender specific cut off points provided by Cole et al. [20]) was determined. Because parents were also asked to report their postal code in the questionnaire, the survey data could be coupled to the neighborhood observation data described in the next paragraph.

### Neighborhood observations

Neighborhoods were selected for observation based on 1) the number of respondents included in the survey living in the neighborhood in order to maximize the number of respondents in the analyses and, 2) physical neighborhood characteristics (based on a neighborhood typology score from the Ministry of Housing, Spatial Planning and the Environment which classifies neighborhoods into the following six categories: city centre, city non-centre, city green, town centre, rural area, and work area [21]) and social neighborhood characteristics (based on the status score from the Netherlands Institute for Social Research which is based on the percentage of immigrants, percentage of people with low education and percentage of low income households per postal code area [22]) in order to maximize the variance in neighborhood characteristics included in the analyses. In total, 57.6% of the parents that filled in a questionnaire during the survey, were living in one of the 33 observed neighborhoods. Hence, combining the data from the survey among parents and the data from the neighborhood observations, resulted in 3,805 individual respondents for the analyses described in this paper.

Data on neighborhood characteristics (the independent variables) were collected by two trained research assistants by means of neighborhood observations in 33 neighborhoods. The observers were not part of the research team to enhance unbiased collection of the data. The two research assistants observed the neighborhoods using a checklist which they completed by mutual agreement. The checklist was based on the Neighborhood Environment Walkability Scale (NEWS) [23], but was specifically adapted for screening Dutch neighborhoods on environmental characteristics related to children’s physical activity [16]. The inter-rater reliability of the checklist was evaluated as good (percentage of agreement = 77%) in a previous Dutch neighborhood observation study [17]. The scoring form included the following seven main topics: 1) buildings (residential density, land use mix, presence of unoccupied houses and maintenance of buildings), 2) formal outdoor play facilities (number and quality of play grounds, school yards, paved play grounds, and half pipe or skating track), 3) public space (presence and quality of green space and water), 4) street pattern (presence and quality of sidewalks and bike lanes) 5) traffic safety (traffic infrastructure and traffic volume and speed), 6) neighborhood characteristics related to the social environment (e.g. street hygiene such as a litter basket for dog waste, graffiti and vandalism (indicating area deprivation) and the presence of a dog walkers area or adequate street lighting which may contribute to social safety) and 7) general impression of the activity-friendliness of the neighborhood for children.

Neighborhood boundaries were defined by local data bases from the municipal organization, so that the results of the study could be easily interpreted by local policy makers. In general, these boundaries correspond with what people perceive as “their neighborhood” and boundaries often coincide with physical “boundaries” such as a railway, busy road, channel or tunnel. In the Netherlands, parents are free to choose a primary school for their child, according to their own opinion and beliefs. Due to practical considerations, many parents choose a primary school close to their home. Hence, in the majority of cases, both the residence and the school of the children will fall within the same neighborhood. Indeed, from our data sample it appeared that 76.5% of the children included in the neighborhood observations, attended school in the same neighborhood as they live in. In cases where the school did not fall within the neighborhood observation area, children were likely to attend school in the adjacent neighborhood.

Similar to another Dutch neighborhood observation protocol developed by Van Lenthe et al. [24], before the start of the actual data collection, a random sample of ten percent of the streets within each neighborhood was selected for observation by foot, based on a list of all streets per neighborhood. Thereafter, all remaining streets in the neighborhood were observed per bicycle, so that all streets were included in the observation. All observations were carried out during normal school days after school time and before dark, to mimic best the real conditions under which children are usually involved in outdoor play in their neighborhood.

### Measures

The dependent variable in all analyses was outdoor play in minutes per week which was calculated by multiplying the number of school days and weekend days the parents reported their child was involved in outdoor play by the average minutes per day the child was involved in outdoor play during school days and weekend days (which was recoded as follows: less than 30 minutes per day = 15 minutes per day, 30 minutes to one hour per day = 45 minutes per day, one to two hours per day = 90 minutes per day, more than two hours per day = 150 minutes per day). Finally, to calculate the total minutes of outdoor play per week, minutes spent on outdoor play during school days and during weekend days were summed.

As stated in the previous paragraph, the neighborhood observation checklist included seven main topics, which yielded in total 33 independent variables which will be described briefly here. A detailed description of all variables included in the analyses is given in Additional file 1: Appendix A. Residential density was estimated by weighting and summing nine items on type of residences in the neighborhood, with a higher sum score representing higher residential density. Land use mix was defined as the proportion of enterprises to residences (range 0-100%). Presence of unoccupied houses was measured on a five-point scale (none-all) and maintenance of buildings was measured on a three-point scale (bad, moderate, good). The total number of play grounds, school yards, paved play grounds, and half pipe or skating track per km^2^ was calculated and summed for each neighborhood, resulting in one score for number of formal outdoor play facilities per km^2^ per neighborhood. Quality of play grounds, school yards, paved playgrounds, and half pipe or skating track was defined on a scale from 0.00 to 1.00, according to ten quality aspects mentioned in Additional file 1: Appendix A. For each type of outdoor play facility separately, 0.10 point per quality aspect was awarded whenever applicable. Hence the quality score per outdoor play facility could range from 0.00 to 1.00, higher scores represent better quality. A mean quality score for all outdoor play facilities per neighborhood was then calculated. Presence of green space, water, sidewalks and bike lanes were each measured on a four-point scale (none-many). Quality of green space, water, sidewalks and bike lanes were defined on a scale from 0.00 to 1.00 (see Additional file 1: Appendix A for specification of quality aspects). Traffic infrastructure included the following single-item variables each measured on a four-point scale (none-many): pedestrian crossings *without* traffic lights, pedestrian crossings *with* traffic lights, traffic lights, refuges / safety islands, parallel parking places, parking lots (grouped), speed bumps, home zones, 30 km/ hour zones, roundabouts, and intersections. Traffic volume and speed was calculated as a sum score of 6 items each measured on a four-point scale (none-many) (Cronbach’s alpha = 0.898), with a higher score representing higher traffic volume and speed. Presence of a dog walking area was a dichotomous item, as was the presence of a litter basket for dog waste and the presence of street lighting. The presence of graffiti, vandalism and dark spaces were each measured on a four-point scale (none – many). General impression of the activity-friendliness of the neighborhood for children was estimated by a score ranging from 1–10, with a higher score representing a more favorable impression. Two items were removed from the analyses due to lack of variation among neighborhoods: the presence of parking garages and the presence of low-traffic / car-free zones. Except for the number of play facilities (which were counted in each neighborhood), the research assistants that performed the neighborhood observations had to give one overall neighborhood score for each of the measures.

### Statistical analyses

From the 3,805 individual respondents in this study, 52 questionnaires were excluded from further analyses because of missing values on the outcome measure outdoor play, and 91 additional questionnaires were removed because of missing values on potential confounders: age or gender of the child (n = 6) and parental education (n = 85). Furthermore, questionnaires of children living more than three days per week on another address than the address described in the questionnaire (n = 18) were removed. Since some questionnaires had to be removed because of more than one exclusion criterion, the final data base for the analyses on outdoor play encompassed 3,651 respondents.

Because different environmental characteristics are expected to be associated with outdoor play between boys and girls and children of different age groups [4], analyses were conducted separately for boys and girls and in age groups 4–6, 7–9, and 10–12 years. Descriptive analyses were conducted with SPSS 17.0 (Chicago, Illinois). ANOVA and chi-square tests were performed to assess differences (p < 0.05) in characteristics between boys and girls in each age group for continuous and categorical variables respectively. Likewise, t-tests and chi-square tests were performed to asses differences (p < 0.05) between respondents that were included in a neighborhood observation (n = 3,805) and the original sample of parents derived from the questionnaire (n = 6,601).

To quantify the association between neighborhood characteristics and children’s outdoor play, multilevel GEE analyses were conducted with SAS 9.1 (Cary, North Carolina). Because most of the independent variables were collected at the neighborhood level, but the dependent variable was collected at the individual level, multi-level analyses with neighborhood as a clustering variable were applied in order to correct for the multi-level structure of the data. Because data were collected completely anonymously, there was no information available on the number of children per household, and hence family-related clustering effects cannot be accounted for in the multi-level analyses. To estimate the possible design effects associated with family clusters, design effect calculations were performed, using the following equation: design effect = 1 + ICC*(n – 1), whereby n is the median number of children within one family. Although municipal data showed that the median number of children within one family was one (which always yields a design effect of 1.00), the calculations were also performed for a median number of two children per family. A pedometer based study by Jacobi et al. [25] has shown that the ICC for physical activity of siblings within one family can be as high as 0.3, hence an ICC of 0.3 is used for the design effect calculations.

Because of the non-normal distribution of the dependent variable outdoor play and its error terms (as assessed by histograms and normal probability plots, data not shown) and since this outcome measure is a count variable (number of minutes outdoor play per week), a Poisson distribution was applied [26,27]. As a consequence, exponents of the original regression coefficient estimates were calculated and interpreted as relative rates (RR). The RR can be interpreted as estimated proportional difference in the amount of outdoor play. For example, an RR of 1.10 indicates 10% longer outdoor play for each additional unit in predictor variables. Due to the Poisson analysis, the proportion of explained variance cannot be reported.

The first step in the analyses focused on environmental characteristics within each of the seven main topics included in the neighborhood observations: buildings, formal outdoor play facilities, public spaces, street pattern, traffic safety, neighborhood characteristics related to the social environment and general impression of the activity-friendliness of the neighborhood for children. All independent variables of one topic were entered simultaneously into a separate model (so one model per topic), which was adjusted for age of the child and parental education level, as indicated by highest completed education of the parent who filled in the questionnaire (it was assumed that this person was the primary caregiver, in the majority of cases this was either the biological mother or the biological father, 81.8% and 11.6% respectively). Parental education level is considered a good indicator for socio-economic status in The Netherlands [28] and is preferred when statistically controlling for socio-economic status in a regression model [29]. Quantitative (i.e. presence or amount) and qualitative aspects of neighborhood characteristics were entered simultaneously in each step of the analyses.

In order to quantify the association between the environmental characteristics and outdoor play when adjusted for the environmental characteristics from other topics, multivariate regression analyses were also performed. In these analyses, all significant (p-value < 0.05) variables from the analyses per topic were entered into a multivariate model, which was also adjusted for age of the child and parental education level. Non-significant variables (p-value > 0.05) were removed one-by-one from the multivariate models (backward elimination procedure), until all variables were statistically significant (p-value < 0.05, except for the potential confounders age of the child and parental education level which were forced into the multivariate model irrespective of significance). In order to check whether choosing a more liberal p-value of 0.10 as a decision criterion for this backward elimination procedure would have influenced the final multivariate models, additionally, all analyses were re-run with a more liberal p-value of 0.10 (same procedure as stated above, only with a p-value of 0.10 instead of 0.05. These additional analyses were performed to check whether potentially important variables were excluded too easily from the multivariate models when applying a p-value of 0.05.

## Results

### Characteristics of the study population

The characteristics of the study population are summarized in Table 1. The study included 1,849 boys and 1,802 girls with an average age of 7.8 years. The average time spent on outdoor play was 411 minutes per week. There were no significant differences in characteristics between boys and girls of the same age groups, except for time spent on outdoor play, which was significantly higher for boys compared to girls in the age groups 7–9 and 10–12 years (p-value = 0.002 and 0.003 respectively).

**Table 1:** **Characteristics of the study population **^**a **^

	**Age 4–6 years**^**b**^		**Age 7–9 years**		**Age 10–12 years**^**b**^		**Total (n = 3,651)**
	**Boys (n = 637)**	**Girls (n = 611)**	**Boys (n = 692)**	**Girls (n = 665)**	**Boys (n = 520)**	**Girls (n = 526)**	
Age (years)	5.0 (0.8)	5.0 (0.8)	8.0 (0.8)	8.0 (0.8)	10.7 (0.7)	10.7 (0.7)	7.8 (2.4)
BMI ^c^ (kg/m^2^)	15.4 (2.0)	15.2 (2.2)	16.3 (2.7)	16.4 (2.7)	17.5 (3.2)	17.4 (2.8)	16.3 (2.7)
Overweight ^d^ (%)	6.5	10.2	11.2	12.9	10.6	11.7	10.5
Obesity ^d^ (%)	3.4	3.8	3.1	4.3	2.7	1.3	3.2
Parental education level (%)							
- Low ^e^	25.3	26.4	29.5	28.1	35.0	35.6	29.6
- Intermediate ^f^	36.6	40.6	36.4	37.4	33.3	31.9	36.2
- High ^g^	38.1	33.1	34.1	34.4	31.7	32.5	34.1
Net household income (Euros per month)	2,754 (1,230)	2,818 (1,277)	2,814 (1,303)	2,761 (1,326)	2,720 (1,345)	2,596 (1,289)	2,751 (1,295)
Outdoor play (minutes per week)	408 (266)	378 (256)	455 (289)*	398 (272)*	444 (284)*	381 (285)*	411 (277)

^a^ Values are mean (SD), unless otherwise specified.

^b^ In The Netherlands, children aged 4–12 years are educated at the same primary school. In the current study sample, two children in the lowest age groups were aged 3 years and 8 children in the highest age groups were aged 13 years. These children were included in the lowest (4–6 years) and highest (10–12 years) age groups, respectively.

^c^ Based on parental self report of height and weight of their child.

^d^ Based on age and gender specific cut off points as provided by Cole et al. [20].

^e^ No education, primary education, lower general secondary education or lower vocational education.

^f^ Higher general secondary education, pre-university education or intermediate vocational education.

^g^ Higher vocational education or university.

* Boldface indicates significant differences between boys and girls within each age group (p < 0.05).

Respondents included in this study were not different compared to the original sample derived from the questionnaire among parents with respect to gender, age, and BMI of the child, percentage of overweight and obese children, and amount of time spent on outdoor play, except for parental education level and net household income, which were significantly lower among the respondents included in the neighborhood observations. Design effect calculations assuming an ICC of 0.3 and a median of one child per family (as suggested by the data from the included municipalities) yielded a design effect of 1.00, whereas assuming a median of two children per family yielded a design effect of 1.30.

Regarding neighborhood characteristics, the size of the 33 included neighborhoods varied from 0.2 to 2.4 km^2^ and the number of inhabitants ranged from 3,175 till 11,407 inhabitants per km^2^. The number of children aged 0–14 years ranged from 449 till 2,000 children per km^2^ and the percentage of children as part of the total number of inhabitants per neighborhood ranged from 11% till 30%.

### Environmental correlates of outdoor play

Table 2 shows the association between neighborhood characteristics and outdoor play as derived from the multivariate analyses for each subgroup of the study population. Due to space limitations, the analyses per topic are not shown, but these can be retrieved from the corresponding author on request.

**Table 2:** **Association between neighborhood characteristics and outdoor play: multivariate analyses **^**a **^

	**Possible range**	**Age 4–6 years**^**b**^		**Age 7–9 years**		**Age 10–12 years**^**b**^	
		**Boys (n = 637)**	**Girls (n = 611)**	**Boys (n = 692)**	**Girls (n = 665)**	**Boys (n = 520)**	**Girls (n = 526)**
***Confounders***							
Age (years)	3-13 ^b^	-	-	-	-	-	-
Parental education level	1-8	-	-	0.95 (0.93-0.98)	0.94 (0.91-0.97)	0.94 (0.92-0.96)	0.96 (0.93-1.00)
***Buildings***							
Residential density	198-368 ^c^	-	**-**	**-**	**-**	**-**	**-**
Land use mix	0-100%	-	-	-	-	-	-
Presence of unoccupied houses	0-4	-	-	-	-	-	-
Maintenance of houses	1-3	-	-	-	-	0.88 (0.83-0.93)	
***Outdoor play facilities***							
Number of formal outdoor play facilities per km^2^	1.99 – 51.85 ^c^	-	-	0.99 (0.99-1.00)	0.99 (0.98-0.99)	0.99 (0.99-1.00)	0.99 (0.98-1.00)
Quality of formal outdoor play facilities	0.00-1.00	-	-	-	-	-	-
***Public space***							
Presence of green space	0-3	-	-	-	-	-	-
Quality of green space	0.00-1.00	-	-	-	-	-	-
Presence of water	0-3	-	-	-	-	-	-
Quality of water	0.00-1.00	-	-	-	-	-	-
***Street pattern***							
Presence of sidewalks	0-3	1.44 (1.16-1.18)	1.66 (1.39-1.99)	-	-	-	1.45 (1.05-2.01)
Quality of sidewalks	0.00-1.00	-	-	-	-	-	-
Presence of bike lanes	0-3	-	-	-	-	-	-
Quality of bike lanes	0.25-1.00	-	-	-	-	-	-
***Traffic safety***							
Presence of pedestrian crossings *without* traffic lights	0-3	-	1.14 (1.01-1.28)	1.20 (1.11-1.29)	-	-	-
Presence of pedestrian crossings *with* traffic lights	0-3	1.13 (1.08-1.19)	-		0.79 (0.67-0.92)	-	-
Presence of traffic lights	0-3	-	-	-	1.48 (1.28-1.72)	-	-
Presence of refuges / safety islands	0-3	-	-	0.89 (0.85-0.93)	-	0.96 (0.93-1.00)	-
Presence of parallel parking places	0-3	-	-	-	-	1.17 (1.07-1.28)	-
Presence of parking lots (grouped)	0-3	-	-	1.28 (1.18-1.38)	-	-	-
Presence of speed bumps	0-3	-	-	1.25 (1.13-1.37)	-	-	-
Presence of home zones	0-3	1.06 (1.02-1.11)	-	-	-	-	-
Presence of 30 km/ hour zones	0-3	-	-	0.82 (0.76-0.89)	-	0.91 (0.86-0.97)	-
Presence of roundabouts	0-3	1.14 (1.07-1.22)	-	1.15 (1.06-1.24)	1.12 (1.01-1.25)	1.10 (1.04-1.16)	-
Presence of intersections	0-3	0.82 (0.74-0.91)	0.78 (0.66-0.91)	0.81 (0.73-0.90)	0.78 (0.69-0.88)	0.87 (0.79-0.97)	-
Traffic volume and speed	0-18	-	-	-	-	-	-
***Social environment***							
Presence of dog walking area	0-1	-	-	-	-	-	-
Presence of litter basket for dog waste	0-1	-	-	-	-	-	-
Presence of graffiti	0-3	-	-	-	-	-	-
Presence of vandalism	0-3	-	-	-	-	-	-
Presence of street lighting	0-1	0.78 (0.97-0.86)	-	-	-	-	-
Presence of dark spaces	0-3	-	-	-	-	-	-
***General impression***							
General impression	1-10	-	-	-	-	-	-

^a^ Values are relative rates (95% confidence intervals). Only significant associations are shown (p-value < 0.05), if the confidence interval contains the value 1.00, this was due to rounding off.

^b^ In The Netherlands, children aged 4–12 years are educated at the same primary school. In the current study sample, two children in the lowest age groups were aged 3 years and 8 children in the highest age groups were aged 13 years. These children were included in the lowest (4–6 years) and highest (10–12 years) age groups, respectively.

^c^ For the variables residential density and number of outdoor play facilities per km^2^, the actual range instead of the possible range is shown.

In the multivariate models, parental education level was negatively associated with outdoor play in the two highest age groups. The relative rates ranged from 0.94 to 0.96 between boys and girls in these two age groups. With regard to the topic “buildings”, the maintenance of houses in the neighborhood was negatively related to outdoor play among boys aged 10–12 years (RR = 0.88). Within the topic “formal outdoor play facilities” the number of formal outdoor play facilities per km^2^ was negatively related to outdoor play in four out of six subgroups (RR = 0.99 in each subgroup), whereas the quality of formal outdoor play facilities was unrelated to outdoor play in all subgroups. None of the variables included in the topic “public space” were significantly related to outdoor play in any of the subgroups. Within the topic “street pattern” the presence of sidewalks showed a positive association with outdoor play among boys aged 4–6 years (RR = 1.44), girls aged 4–6 years (RR = 1.66) and girls aged 10–12 years (RR = 1.45). Several variables within the topic “traffic safety” were positively related to outdoor play in the different subgroups included in this study: the presence of pedestrian crossings *without* traffic lights (e.g. zebra crossings) among girls aged 4–6 years (RR = 1.14) and boys aged 7–9 years (RR = 1.20), the presence of pedestrian crossings *with* traffic lights among boys aged 4–6 years (RR = 1.13), the presence of traffic lights among girls aged 7–9 years (RR = 1.48), the presence of parallel parking spaces among boys aged 10–12 years (RR = 1.17), the presence of grouped parking lots among boys aged 7–9 (RR = 1.28), the presence of speed bumps among boys aged 7–9 years (RR = 1.25), and the presence of home zones among boys aged 4–6 years (RR = 1.06). Other traffic safety items were negatively associated with outdoor play: the presence of pedestrian crossings *with* traffic lights, the presence of refuges or safety islands among boys aged 7–9 years and boys aged 10–12 years (RR = 0.89 and RR = 0.96 respectively), and the presence of 30 km / hour zones among boys in the highest two age groups (RR = 0.82 and 0.91 for boys aged 7–9 and 10–12 years respectively). In general, the presence of roundabouts was positively associated with outdoor play (RR ranged from 1.10 to 1.15 in four out of six subgroups), whereas the presence of intersections was negatively associated with outdoor play (RR ranged from 0.78 to 0.87 in five out of six subgroups). Traffic volume and speed was not significantly related to outdoor play in any of the subgroups. None of the variables included in the topic “neighborhood characteristics related to the social environment” were significantly related to outdoor play in any of the subgroups, except for street lighting, which showed a negative association with outdoor play among boys age 4–6 years (RR = 0.78). Likewise, the general impression of activity-friendliness of the neighborhood for children was not significantly related to outdoor play. Rerunning the analyses with a p-value of 0.10 did not drastically alter the results (data not shown).

## Discussion

This study showed that, apart from individual factors such as parental education level, certain modifiable characteristics in the neighborhood environment (as measured by neighborhood observations) were associated with outdoor play among boys and girls of different age groups in The Netherlands. The finding that parental education level was negatively associated with outdoor play, might be explained by the fact that higher educated parents have more financial resources for organized sports activities, and that this substitutes time spent on outdoor play [30]. Moreover, as lower educated parents might live in smaller houses, this makes it more likely for children to play outdoors. Another explanation might be found in the finding that parents living in more socioeconomic deprived areas are more likely to allow their children to take part in outdoor activities independently [31]. Veitch et al. recently have shown that the correlation between parental education level and the time spent on outdoor play, is different for different outdoor play locations, i.e. children of higher educated parents are more likely to play in the private yard at home, but are less likely to play in their own street, in a park or on a play ground [32].

In contrary with the expectation, the number of formal outdoor play facilities showed a small, but significant, negative association with outdoor play among four out of six subgroups whereas the overall quality of formal outdoor play facilities was unrelated to outdoor play. The disadvantage of taking number of play facilities per km^2^ as an indicator for “quantity of play facilities” and calculating an average score for “quality of outdoor play facilities”, is that it ignores the size and quality of each individual play facility as a possible important factor in relation to children’s outdoor play behavior, which might be concentrated around the play facilities most close to their homes. Therefore, future research should aim to measure the size and quality of each individual play facility and this should further elucidate the exact correlation between presence and quality of individual outdoor play facilities in proximity to children’s homes.

On the other hand, the presence of sidewalks and parallel or grouped parking places was positively associated with outdoor play in three subgroups. This might indicate that in The Netherlands “informal” play areas such as sidewalks might be more important in relation to outdoor play than the formal play facilities such as playgrounds or school yards. This hypothesis is in line with other Dutch research using neighborhood observations, which suggests that the presence of parallel parking places might serve as an informal place to play, or could function as a barrier between children playing on the sidewalks and cars on the road [16]. The fact that the positive association between sidewalks and outdoor play was found in the lowest age group (both boys and girls) suggests that especially for younger children, sidewalks provide for an informal play space close to their home, suitable for outdoor play activities such as rope skipping, hopscotch or skating.

Features of the public space (i.e. presence and quality of green space and water), characteristics of the buildings in the neighborhood, neighborhood characteristics related to the social environment and the general impression of activity-friendliness of the neighborhood for children were mostly unrelated to outdoor play. USA accelerometer and GIS data among overweight children however, did find a positive association between parks in the neighborhood and children’s physical activity [33]. UK data on the other hand showed that most of children’s outdoor physical activity occurs in non-green urban environments [34], which is in line with the finding from the present study that sidewalks may provide for an important outdoor play opportunity. These discrepancies underline the difficult comparison between research results from the USA vs. Europe and between studies that utilize different methods and outcomes measures. Items included in the walkability concept (such as land-use mix and residential density) have been shown to be of importance in relation to active commuting to school [35], but the results of the current study do not point to an important role for those walkability items in relation outdoor play among Dutch children, except for the presence of sidewalks, which might be part of the walkability concept as well. Holt et al. even suggest that “low-walkable” neighborhoods (with for example many cul-de-sacs) are more beneficial for younger children to get involved in outdoor play [36]. Taken together, these findings suggest that walkability is a different concept than “playability” and that these concepts should both be taken into account when designing activity-friendly neighborhoods.

Furthermore, Giles-Corti et al. argue that traffic safety should be included into the walkability concept when applied to children’s physical activity behavior [35]. Items within the topic traffic safety indeed did show significant associations with outdoor play in the current study, although there was some variation across subgroups (more specifically, traffic safety items were related to outdoor play particularly among boys). The presence of pedestrian crossings or traffic lights was positively associated with outdoor play as was the presence of parking places. The fact that some traffic items such as the presence of refuges / safety islands were negatively associated with outdoor play, may reflect the fact that these infrastructural facilities are usually present at busy streets. Quite consistently among all subgroups, we found a negative association between the presence of intersections and outdoor play on the one hand, and a positive association between the presence of roundabouts and outdoor play on the other hand. Together with results from other studies that demonstrate the importance of parental (traffic) safety concerns [37], this might be a valuable finding for policy makers within sectors such as spatial planning and traffic and transportation, when (re)designing neighborhoods that are activity-friendly for children. Because the influence of road safety on children’s physical activity level is dependent on age, gender and type of physical activity [38-40] it remains important to pay attention to these differences and the local neighborhood context. Ideally, a neighborhood should contain appropriate opportunities for play for boys and girls of different age categories.

Neighborhood characteristics related to the social environment were not related to children’s outdoor play behavior in this study. This is in contrary to a previous study among the same study population using subjective methods (i.e. written questionnaires for parents) to quantify the perceived environmental characteristics related to outdoor play among children [10]. In the previous study, perceived social neighborhood characteristics such as social cohesion were consistently (and positively) related to outdoor play. Social cohesion however, is a different concept than the neighborhood characteristics related to the social environment as measured with the observation protocol in this study, which might be an explanation for the discrepancies between the two studies. For example, the social cohesion measure in our previous study included items about the values, norms, and trust prevailing among neighborhood residents, and those concepts are difficult to measure by means of neighborhood observations. Another possible explanation may lie in the differences between parent’s perception of the immediate environment that children have access to around their residence and the overall neighborhood characteristics as measured by neighborhood observations. Future research should address the relative importance of both concepts in relation to children’s age, because children’s action radius is enlarged with increasing age. Furthermore, these results indicate that it is important to change both the actual neighborhood characteristics, as well as the perception of these characteristics by people (parents as well as children) living in that neighborhood.

Although the general impression of the activity-friendliness of the neighborhood was shown to be related to moderate to vigorous physical activity among children in a previous Dutch neighborhood observation study [16], the general impression of the activity-friendliness was not related to a specific component of physical activity (outdoor play) in this study. Once again, this underlines the need for specificity in studying the relation between environmental characteristics and components of physical activity behavior [18].

Regarding the study design, some limitations should be mentioned. Due to the cross-sectional design of the study, no causal relations can be demonstrated. However, because we derived the outcome measure and the neighborhood characteristics from different data sources, same source bias was prevented [41]. Although the questionnaires were administered one year earlier than the neighborhood observations, it is unlikely that the neighborhood characteristics have changed within the time span of one year. Moreover, although there is a large overlap between the place where children live, where they go to school, and where we performed our neighborhood observation, the actual place where children are involved in outdoor play could be somewhere else (at a friend’s home for example), and this cannot be derived from the data gathered in this study. Future research using GPS technique could provide more insight into the actual location where children play.

A major shortcoming of the neighborhood observation technique used in this study is that some neighborhood characteristics, for example the quality of play facilities may vary within one neighborhood. The research assistants that carried out the neighborhood observations reported that in neighborhoods with more than one play facility, the quality of those facilities could vary. Especially for larger neighborhoods with more than one play facility, it may be necessary to conduct additional research to reveal the exact location where children (living in different parts of the same neighborhood) play, and whether this correlates with the quality and location of the different play facilities.

Although the questions on physical activity were not validated, the questions were derived from a standard questionnaire used for monitoring purposes in the Netherlands [42]. Because of the large scale set up of the study, it was not possible to measure children’s physical activity level more objectively (e.g. by use of accelerometers). Moreover, accelerometers cannot give information about the amount of time spent on specific types of physical activity (such as outdoor play, sports participation or active commuting), whereas these different types of physical activity are presumably associated with different environmental characteristics [18].

Poisson regression analysis was applied to overcome the violation of assumption of normality in the dependent variable. This was possible because the outcome variable outdoor play was defined as the total number of minutes per week the child was involved in outdoor play. However, the questions underlying the calculation of the total number of minutes involved in outdoor play were categorical, which may have introduced error in the measurements. In addition, although multi-level analyses were applied for clustering within neighborhoods, the analyses were not adjusted for clustering within households (i.e. children within households, when parents filled in a questionnaire for more than one child). Additional design effect calculations however show that with an assumed ICC of 0.3 for children within one family and a median number of one or two children per family, the design effect for clustering within families is 1.00 and 1.30 respectively. In the latter case this would mean that the power of our study would be lower than expected as based on our initial power calculations and the confidence intervals of our estimates would be wider. Lastly, because data were collected in four medium sized cities in the South of The Netherlands, results can only be generalized to other cities with a comparable size and population. The results are particularly suitable for underpinning local policy measures in the four participating cities.

## Conclusions

In conclusion, the results show that the quantity and overall quality of formal outdoor play facilities were not positively related to outdoor play among children in this study. Rather, informal play areas such as the presence of sidewalks were related to children’s outdoor play. Also, traffic safety was an important characteristic associated with outdoor play. Local policy makers from different sectors can use these research findings in creating more activity-friendly neighborhoods for children.

## Competing interests

The authors declare that they have no competing interests.

## Authors’ contributions

MJA coordinated the data collection, performed the data analysis and drafted the manuscript. SIdV provided the neighborhood observation protocol and critically commented on the data analysis and drafts of the manuscript. JAMvO and AJS supervised the data collection and data analysis and critically revised the manuscript. All authors read and approved the final manuscript.

## Supplementary Material

Additional file 1 **Appendix A.** Overview of the dependent and independent variables included in the analyses. Click here for file
