# Supplementary material for: Outdoor play among children in relation to neighborhood characteristics: a cross-sectional neighborhood observation study
Source: Int J Behav Nutr Phys Act. 2012 Aug 17;9:98. doi: 10.1186/1479-5868-9-98 (PMC3478217; doi:10.1186/1479-5868-9-98)
Supplement: Additional file 1 — Appendix A. Overview of the dependent and independent variables included in the analyses. [file 1479-5868-9-98-S1.docx]

Appendix A: Overview of the dependent and independent variables included in the analyses

| **Concept** | **Number of items** | **Exact formulation of items** | **Cronbach’s**  **alpha** | **Answer categories** | **Recode / sum score calculation and range** | **Descriptive data: mean (SD) or proportions** |
| --- | --- | --- | --- | --- | --- | --- |
| Dependent variable (questionnaire among parents) | | | | | | |
| Outdoor play | 4 | Considering a typical week in the past month:  How many days does your child play outdoors on school days?  On average, how long does your child spend on outdoor play on such a weekday?  How many days does your child play outdoors during the weekend?  On average, how long does your child spend on outdoor play on such a weekend day? | NA | For the first item: 0 or less than 1 day per week, 1 day per week, 2 days per week, 3 days per week, 4 days per week, 5 days per week.  For the second and fourth item: my child does not play outside during weekdays / weekend days, less than 30 minutes per day, 30 -60 minutes per day, 60-120 minutes per day, more than 120 minutes per day.  For the third item: 0 or less than 1 day per weekend, 1 day per weekend, 2 days per weekend. | The minutes per day the child was involved in outdoor play was recoded as follows: my child does not play outside = 0 minutes per day, less than 30 minutes per day = 15 minutes per day, 30 minutes to one hour per day = 45 minutes per day, one to two hours per day = 90 minutes per day, more than two hours per day = 150 minutes per day. Number of days involved in outdoor play on school days / weekend days was multiplied by the minutes spent on outdoor play per school day / weekend day. Minutes spent on outdoor play during school days and weekend days were summed to give the total minutes of outdoor play in a seven day week. | 411 (277) minutes per week |
| Individual factors (questionnaire among parents) | | | | | | |
| Gender of the child | 1 | What is the gender of your child? | NA | Boy; girl. | NA | 50.6% boy; 49.4% girl. |
| Age of the child | 1 | How old is your child? | NA | Open question | Range from 3 to13 years. ^a^ | 7.8 (2.4) years |
| Parental education | 1 | What is your highest completed education? | NA | No education; primary education; lower vocational education; lower general secondary education; intermediate vocational education; higher general secondary education or pre-university education; higher vocational education; university. | Parental education was treated as an ordinal variable ranging from 1 to 8, higher scores represent higher education. | 4.3% no education; 2.8% primary education; 11.7% lower vocational education, 10.8% lower general secondary education; 25.6% intermediate vocational education; 10.7% higher general secondary education or pre-university education; 24.0% higher vocational education; 10.1% university. |
| Buildings (neighborhood observations) | | | | | | |
| Residential density | 9 | How often do the following types of residences occur in the neighborhood? | NA | Detached residences; semi-detached / duplex residence; row houses / single-family dwelling; upstairs flat / maisonette; flats / condo’s < 3 stories; flats / condo’s 4-6 stories; flats / condo’s < 6-12 stories; flats / condo’s > 12 stories; farmhouses. Each type of residence was scored on a five-point Likert-type scale ranging from 1 (none) to 5 (all). | The scores were weighted and summed to calculate residential density (higher scores represent greater density), according to the protocol of Saelens et al. (23). | 247 (45) |
| Land use mix | 1 | What is the proportion of enterprises to residences? | NA | 0-100% | NA | 15.2 (14.7) |
| Presence of unoccupied houses | 1 | How many unoccupied houses are there in the neighborhood? | NA | Five-point Likert-type scale (none-all). | Range from 0-4, higher score represent more unoccupied houses. | 0.1 (0.3) |
| Maintenance of buildings | 1 | How is the maintenance of buildings in the neighborhood? | NA | Three-point Likert-type scale (bad -good). | Range from 1-3, higher scores represent better maintenance. | 2.7 (0.6) |
| Formal outdoor play facilities (neighborhood observations) | | | | | | |
| Number of formal outdoor play facilities per km^2^ | 4 | How many playgrounds, schoolyards, paved playgrounds and half pipes or skating racks are there in the neighborhood? | NA | Open question. | The total number of outdoor play facilities in the neighborhood was divided by the number of km^2^ per neighborhood. | 12.8 (8.4) |
| Quality of formal outdoor play facilities | 10 | Ten quality aspects of each outdoor play facility were scored. | NA | 1) location of play facility nearby residences, 2) safely accessible, 3) always accessible, 4) sufficient surveillance during daytime, 5) good level of maintenance, 6) sufficient (street) lighting, 7) sufficient variation in outdoor play equipment, 8) useful separation from the street (e.g. a fence, covering), 9) absence of trash and litter, and 10) absence of dog waste. | Each quality aspect was awarded with 0.10 point whenever applicable, for each type of outdoor play facility separately. Hence the total quality score per outdoor play facility could range from 0.00 to 1.00, higher scores represent better quality. A mean quality score for all outdoor play facilities per neighborhood was then calculated. | 0.7 (0.1) |
| Public space (neighborhood observations) | | | | | | |
| Presence of green space | 1 | How much green space (trees, grass, parks and public green spaces) is there in the neighborhood? | NA | Four-point Likert-type scale (none-a lot). | Range from 0 to 3, higher scores represent more green space. | 1.5 (0.8) |
| Quality of green space | 4 | Four quality aspects of green space were scored. | NA | 1) good level of maintenance, 2) absence of weeds and thorn-bushes, 3) absence of trash and litter, 4) absence of dog waste. | Each quality aspect was awarded with 0.25 point whenever applicable. Hence the total quality score could range from 0.00 to 1.00, higher scores represent better quality. | 0.4 (0.2) |
| Presence of water | 1 | How much water (ditches, pools, lakes) is there in the neighborhood? | NA | Four-point Likert-type scale (none-a lot). | Range from 0 to 3, higher scores represent more water. | 1.3 (0.9) |
| Quality of water | 4 | Four quality aspects of water were scored. | NA | 1) good level of maintenance, 2) absence of deep waters, 3) absence of high and steep watersides, 4) absence of trash and litter. | Each quality aspect was awarded with 0.25 point whenever applicable. Hence the total quality score could range from 0.00 to 1.00, higher scores represent better quality. | 0.6 (0.2) |
| Street pattern (neighborhood observations) | | | | | | |
| Presence of sidewalks | 1 | How many sidewalks are there in the neighborhood? | NA | Four-point Likert-type scale (none-a lot). | Range from 0 to 3, higher scores represent more sidewalks. | 2.9 (0.2) |
| Quality of sidewalks | 4 | Four quality aspects of sidewalks were scored. | NA | 1) good level of maintenance, 2) good quality (e.g. width and room to walk), 3) absence of trash and litter, 4) absence of dog dirt. | Each quality aspect was awarded with 0.25 point whenever applicable. Hence the total quality score could range from 0.00 to 1.00, higher scores represent better quality. | 0.9 (0.2) |
| Presence of bike lanes | 1 | How many bike lanes are there in the neighborhood? | NA | Four-point Likert-type scale (none-a lot). | Range from 0 to 3, higher scores represent more bike lanes. | 1.6 (0.6) |
| Quality of bike lanes | 4 | Four quality aspects of bike lanes were scored. | NA | 1) good level of maintenance, 2) good quality (e.g. width and room to bicycle), 3) bike lane integrated in main road by means of a dashed line, 4) bike lane separated from main road by means of a solid line or other separation. | The four quality aspects were awarded with 0.25 or 0.50 points whenever applicable: good level of maintenance (0.25 points), good quality (0.25 points), bike lane integrated in main road by means of a dashed line (0.25 points), or bike lane separated from main road by means of a solid line or other separation (0.50 points). Hence the total quality score could range from 0.00 to 1.00, higher scores represent better quality. | 0.7 (0.3) |
| Traffic safety (neighborhood observations) | | | | | | |
| Presence of pedestrian crossings *without* traffic lights | 1 | How many pedestrian crossings *without* traffic lights are there in the neighborhood? | NA | Four-point Likert-type scale (none-a lot). | Range from 0 to 3, higher scores represent more pedestrian crossings *without* traffic lights. | 0.8 (0.8) |
| Presence of pedestrian crossings *with* traffic lights | 1 | How many pedestrian crossings *with* traffic lights are there in the neighborhood? | NA | Four-point Likert-type scale (none-a lot). | Range from 0 to 3, higher scores represent more pedestrian crossings *with* traffic lights. | 0.6 (0.8) |
| Presence of traffic lights | 1 | How many traffic lights are there in the neighborhood? | NA | Four-point Likert-type scale (none-a lot). | Range from 0 to 3, higher scores represent more traffic lights. | 0.5 (0.7) |
| Presence of refuges / safety islands | 1 | How many refuges / safety islands are there in the neighborhood? | NA | Four-point Likert-type scale (none-a lot). | Range from 0 to 3, higher scores represent more refuges / safety islands. | 1.0 (0.9) |
| Presence of parallel parking places | 1 | How many parallel parking places are there in the neighborhood? | NA | Four-point Likert-type scale (none-a lot). | Range from 0 to 3, higher scores represent more parallel parking places. | 2.9 (0.3) |
| Presence of parking lots (grouped) | 1 | How many parking lots are there in the neighborhood? | NA | Four-point Likert-type scale (none-a lot). | Range from 0 to 3, higher scores represent more parking lots. | 2.2 (0.6) |
| Presence of speed bumps | 1 | How many speed bumps are there in the neighborhood? | NA | Four-point Likert-type scale (none-a lot). | Range from 0 to 3, higher scores represent more speeds bumps. | 2.3 (0.8) |
| Presence of home zones | 1 | How many home zones are there in the neighborhood? | NA | Four-point Likert-type scale (none-a lot). | Range from 0 to 3, higher scores represent more home zones. | 2.1 (0.9) |
| Presence of 30 km/ hour zones | 1 | How many 30 km/ hour zones are there in the neighborhood? | NA | Four-point Likert-type scale (none-a lot). | Range from 0 to 3, higher scores represent more 30 km/ hour zones. | 2.4 (0.7) |
| Presence of roundabouts | 1 | How many roundabouts are there in the neighborhood? | NA | Four-point Likert-type scale (none-a lot). | Range from 0 to 3, higher scores represent more roundabouts. | 0.6 (0.7) |
| Presence of intersections | 1 | How many intersections are there in the neighborhood? | NA | Four-point Likert-type scale (none-a lot). | Range from 0 to 3, higher scores represent more intersections. | 1.3 (0.6) |
| Traffic volume and speed | 6 | How many fast driving cars or motorcycles are there in the neighborhood?  How many fast driving scooters or mopeds are there in the neighborhood?  How many cars are parked outside parking places in the neighborhood?  How much busy traffic is there in the neighborhood?  How much heavy truck or bus traffic is there in the neighborhood?  How many other traffic problems are there in the neighborhood? | 0.898 | Each item was scored on a four-point Likert-type scale (none-a lot). | Range from 0 to 18, higher scores represent higher traffic volume and speed. | 4.7 (3.7) |
| Neighborhood characteristics related to the social environment (neighborhood observations) | | | | | | |
| Presence of dog walking area | 1 | Is there a dog walking area in the neighborhood? | NA | No; yes. | No (0), yes (1). | 51.9 % no; 48.1% yes. |
| Presence of litter basket for dog waste | 1 | Is there a litter basket for dog waste in the neighborhood? | NA | Absent; insufficiently present; sufficiently present. | Absent and insufficiently present were recoded into 0, sufficiently present was coded as 1. | 88.0 % absent or insufficiently present; 12.0 % sufficiently present. |
| Presence of graffiti | 1 | How much graffiti is there in the neighborhood? | NA | Four-point Likert-type scale (none-a lot). | Range from 0 to 3, higher scores represent more graffiti. | 1.6 (0.7) |
| Presence of vandalism | 1 | How much vandalism (e.g. vandalized bus shelters or litter baskets) is there in the neighborhood)? | NA | Four-point Likert-type scale (none-a lot). | Range from 0 to 3, higher scores represent more vandalism. | 0.2 (0.4) |
| Presence of street lighting | 1 | Is there street lighting in the neighborhood? | NA | Absent; insufficiently present; sufficiently present. | Absent and insufficiently present were recoded into 0, sufficiently present was coded as 1. | 3.1 % absent or insufficiently present; 96.1 % sufficiently present. |
| Presence of dark spaces | 1 | How many dark spaces (such as tunnels) are there in the neighborhood? | NA | Four-point Likert-type scale (none-a lot). | Range from 0 to 3, higher scores represent more dark spaces. | 1.4 (0.7) |
| General impression (neighborhood observations) | | | | | | |
| General impression | 1 | What is your general impression of the neighborhood in terms of opportunities to play, walk, bicycle for children? | NA | 1-10 | NA | 7.1 (1.2) |

^a^ In The Netherlands, children aged 4–12 years are educated at the same primary school. In the current study sample, 3 children in the lowest grade were aged 3 years and 8 children in the highest grade were aged 13 years. These children were included in the lowest (4-6 years) and highest (10-12 years) age groups, respectively.

SD = Standard deviation.

NA = Not applicable.
